# Supplementary material for: Pseudogymnoascus destructans Transcriptional Response to Chronic Copper Stress
Source: J Fungi (Basel). 2025 May 13;11(5):372. doi: 10.3390/jof11050372 (PMC12113139; doi:10.3390/jof11050372)
Supplement: Supplementary file 1 [file jof-11-00372-s001.zip › Figures S1 and S2.pdf]

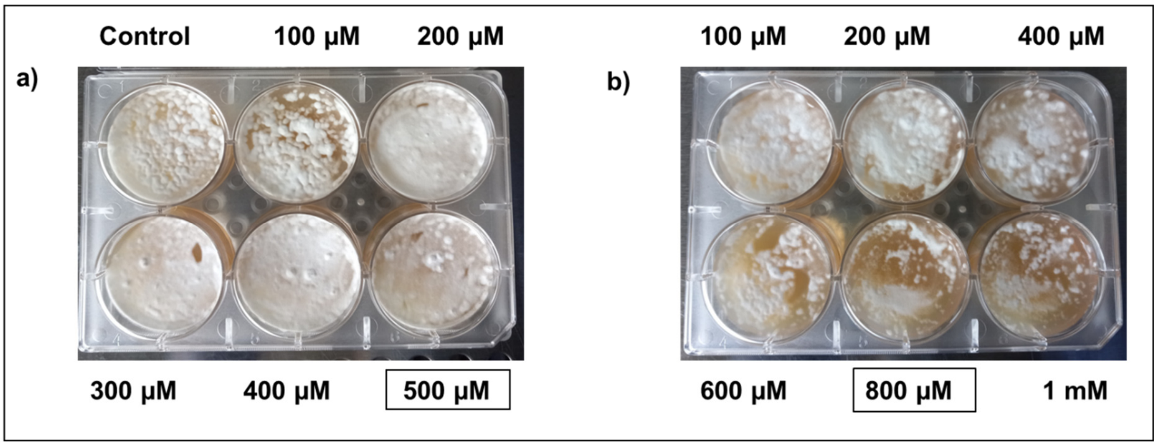

Figure S1. Growth of *Pseudogymnoasus destuctans* on synthetic (Sc-Ura) plates supplemented with $CuSO_4$ or BCS. *P. destuctans* were grown for 6 days under varying concentrations of a) copper and b) BCS stress conditions. Here, we have selected an intermediate concentration of 500 µM $CuSO_4$ to stimulate Cu-overload growth conditions and 800 µM copper chelator BCS to stimulate Cu-withholding growth conditions.

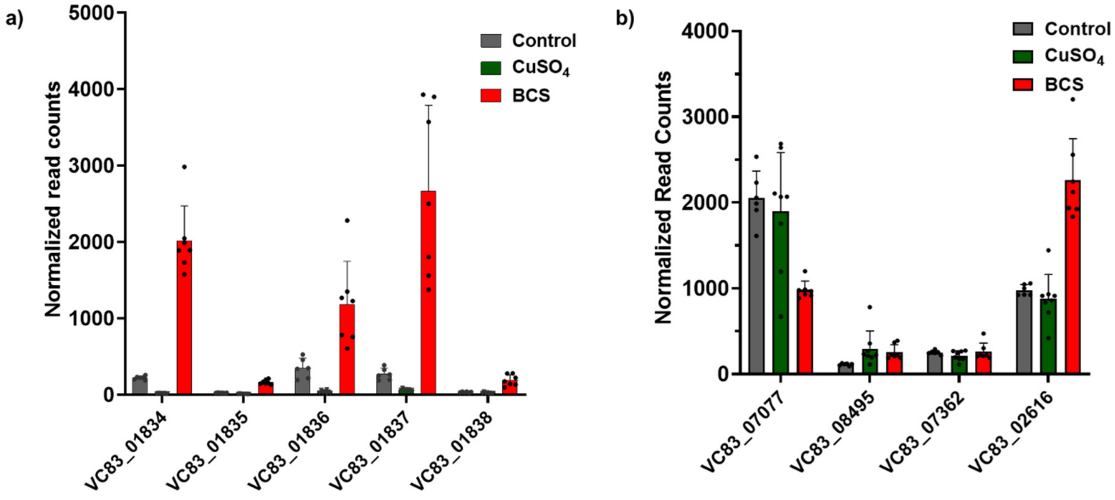

Figure S2. Normalized read counts for *Pseudogymnoasus destuctans* CRC and SOD genes. a) Copper-responsive gene cluster (CRC). b) Superoxide dismutase (SOD) genes. (Control: n = 6, $CuSO_4$: n = 8, BCS: n = 7) ****VC83_08495 is multiplied by 10 for graph clarity.**
