# Supplementary material for: Pseudogymnoascus destructans Transcriptional Response to Chronic Copper Stress
Source: J Fungi (Basel). 2025 May 13;11(5):372. doi: 10.3390/jof11050372 (PMC12113139; doi:10.3390/jof11050372)
Supplement: Supplementary file 1 [file jof-11-00372-s001.zip › File S4.pdf]

File S4. *P. destructans* genome encodes for two Mn-SOD enzymes. VC83_07362 contains an N-terminal mitochondrial localization leader sequence while VC83_02616 lacks this peptide motif and may reside in the fungal cytoplasm.

Clustal omega Sequence alignment of VC83_07362 and VC83_02616. Highlighted in yellow is the putative N-terminal mitochondrial targeting sequence for VC83_07362. There is no predicted mitochondrial localization sequence for VC83_02616. Based on these predictions we propose that VC83_02616 may localize in the cytoplasm. The TargetP-2.0 server found at (https://services.healthtech.dtu.dk/services/TargetP-2.0/) was used for assignments of Mitochondrial localization signal sequences.

```
VC83_07362      MSATLFRISPAVRSALKAGASKRVARVASTSFVRSKATLPDLQYDYGALEPAISGKIMEL      60
VC83_02616      -------------------------------MSSSKYVLPKLPYAYNALEPYISEQIMTI      29
                                               :  ** .**.* * *.**** ** :** :

VC83_07362      HHSKHHQTYVTSYNAATEQFQAAEAKQDIAAKVALQPLINFHGGGHLNHTLFWENLAPKS      120
VC83_02616      HHSKHHQTYVNNLNIALLSQATAVSTNSLAHQINLQTAIRFNAGGHINHALFWGNLTSAA      89
                **********.. * *  .  :* :.:.:* :: **  *.*:.***:**:*** **:  :

VC83_07362      QGGGRE---PSGALKTAIEDSYGSFIDFQGKFNTALAGIQGSGWAWLVKDNQTGKVLIKT      177
VC83_02616      ETAPSPTSSVAPRLVAALESQWGSVQVFKEKFEAALLAIQGSGWGWLVQDVDTQRLEITT      149
                : .       :  * :*:*..:**.  *: **::** .******.***:* :* :: *.*

VC83_07362      YANQDPVVGQYTPILGVDAWEHAYYLQYENRKAEYFKAIWDVLNWKTAEKRF--------      229
VC83_02616      SKDQDIVPKGKKPLLGIDMWEHAYYLQYLNNKKDYAAGIWNVINWTVVEKRLSTDVDVVF      209
                  :** *    .*:**:* ********* *.* :*  .**:*:**...***:

VC83_07362      ----------    229
VC83_02616      NIVGTLGANL    219
```
