# Supplementary material for: Pseudogymnoascus destructans Transcriptional Response to Chronic Copper Stress
Source: J Fungi (Basel). 2025 May 13;11(5):372. doi: 10.3390/jof11050372 (PMC12113139; doi:10.3390/jof11050372)
Supplement: Supplementary file 1 [file jof-11-00372-s001.zip › Table S1.pdf]

Table S1. Functional Annotation and Expression Profile of 7 Gene Cluster Under Copper Stress

**Cluster (7 genes) DR C vs BCS and UR C vs Cu**	**Protein Function or Localization**	**Annotation**
VC83_07925	ND	Zinc finger
VC83_03107	Secreted	Hydrophobic surface binding protein A https://doi.org/10.1128/AEM.72.4.2407-2413.2006
VC83_07926	ND	ND
VC83_06529	Secreted	ND
VC83_08519	MFS	Fungal trichothecene efflux pump (TRI12)
VC83_05770	secreted	Aerolisin/ETX pore-forming domain 1 hit (SSF56973)
VC83_03991	Cell membrane	MARVEL domain-containing protein

* ND not determined
