# Supplementary material for: Pseudogymnoascus destructans Transcriptional Response to Chronic Copper Stress
Source: J Fungi (Basel). 2025 May 13;11(5):372. doi: 10.3390/jof11050372 (PMC12113139; doi:10.3390/jof11050372)
Supplement: Supplementary file 1 [file jof-11-00372-s001.zip › Tables S2 and S3.pdf]

Table S2. Comparative blast analysis of selected significantly regulated genes by Cu-withholding stress (BCS) and their fungal homologs

**Genes**	**Homologs**				**Log_2FC**	**logCPM**	**Cellular Component**	**Biological process**	**Conserved Protein Domain Family**
	Saccharomyces cerevisiae **S288C (taxid:559292)**	***Candida albicans*** **SC5314 (taxid:237561)**	***Aspergillus niger*** **(taxid:5061)**	***Cryptococcus neoformans*** **(taxid:5207)**					
VC83_07909	ND	ND	ND	ND	5.14	7.12	ND	melanin metabolic process	cl09109: Nuclear transport factor 2
VC83_07077	SOD1	SOD1	SOD1	SOD	-1.07	10.53	ND	superoxide metabolic process	pfam00080: Copper/zinc superoxide dismutase
VC83_08495	ND	SOD5	GKZ53660.1	ND	1.20	4.28	TransMembrane:1	superoxide metabolic process	pfam00080:Copper/zinc superoxide dismutase
VC83_02616	SOD2	SOD2, SOD3	KAI2825965.1	SOD2	1.23	10.72	cytosol	superoxide metabolic process	COG0605:Superoxide dismutase
VC83_04976	RGT2	HGT12	GAQ40674.1	OXC63079.1	2.17	8.89	Integral to membrane	carbohydrate transport	pfam00083: Sugar (and other) transporter
VC83_01371	MAL11	MAL31	KAI2883056.1	OXG15676.1	2.27	7.85	Membrane	carbohydrate transport	cl26863:Sugar (and other) transporter
VC83_05061	CCP1	CCP1	KAI2926762.1	OXB37400.1	1.45	8.63	Mitochondrion	response to oxidative stress	cd00691: ascorbate_peroxidase
VC83_00191	ND	ND	TPR06291.1	ND	3.59	11.63	Plasma membrane	copper ion transport	pfam04145:Ctr copper transporter family
VC83_01360	ZRT2	ZRT1	GAQ43504.1	OXG99752.1	1.48	5.59	Plasma membrane	metal ion transport	cl00437: ZIP Zinc transporter
VC83_00187	ND	ND	KAL3250585.1	ND	1.56	3.85	ND	electron transport	cl12078: Cytochrome P450
VC83_07080	CCP1	CCP1	EHA26204.1	OXH31575.1	1.31	7.86	Cytosol	electron transport	cl00196:Heme-dependent peroxidases
VC83_06736	ND	XP_715252.1	KAI3006497.1	OWZ26292.1	1.01	5.73	ND	electron transport	cd04730:2-Nitropropane dioxygenase (NPD)
VC83_08787	FRE5	CFL11	GKZ79616.1	OXH01474.1	5.37	7.88	Plasma membrane	electron transport	cd06186: NADPH oxidase (NOX)
VC83_08524	DIT2	ERG5	KAI3009392.1	OWZ69196.1	3.84	6.97	ND	electron transport	cl12078: Cytochrome P450
VC83_08659	AIM17	KHC85949.1	EHA24645.1	OWZ80585.1	1.54	3.34	Mitochondrion	electron transport	cl26676:Taurine dioxygenase
VC83_08976	BNA4	BNA4	GKZ96846.1	UOH81715.1	-1.35	10.67	ND	electron transport	cl27552:FAD binding domain
VC83_02488	CBR1	CBR1	GAQ41792.1	OXC64979.1	2.16	5.24	ND	electron transport	cl26810:nitrate reductase [NADPH]
VC83_02494	YHB1	YHB1	GLA16543.1	OWT39490.1	4.23	6.91	Intracellular anatomical structure	electron transport	cl26811:NAD(P)H-flavin reductase
VC83_03586	BNA4	BNA4	KAI2848814.1	OWZ67419.1	1.28	7.03	Mitochondrial outer membrane	electron transport	COG0654:UbiH; 2-polyprenyl-6-methoxyphenol hydroxylase and related FAD-dependent oxidoreductases
VC83_03003	ND	ND	EHA20505.1	OXC80805.1	1.02	6.04	ND	electron transport	cd04730:2-Nitropropane dioxygenase (NPD)
VC83_03096	FRE7	FRP1	KAL3253552.1	OWZ43078.1	1.52	10.29	Plasma membrane	electron transport	cd06186:NADPH oxidase (NOX)
VC83_03826	ND	ND	KAI2885967.1	ND	1.21	6.09	ND	electron transport	cl00184:Clavaminic acid synthetase (CAS)
VC83_04399	ND	ND	EHA22966.1	ND	-1.18	5.86	Integral to membrane	electron transport	cd08760: Cyt_b561_FRRS1_like; Eukaryotic cytochrome b(561)

*ND-Not determined

Table S3. Comparative blast analysis of selected significantly regulated genes by Cu-overload stress and their fungal homologs.

**Genes**	**Homolog**				**Log2FC**	**logCPM**	**Cellular component**	**Biological process**	**Conserved Protein Domain Family**
	***Saccharomyces cerevisiae S288C* (taxid:559292)**	***Candida albicans SC5314* (taxid:237561)**	***Aspergillus niger* (taxid:5061)**	***Cryptococcus neoformans* (taxid:5207)**					
VC83_02490	ND	DAL9	EHA18141.1	OXG18647.1	3.60	4.01	Integral to membrane	inorganic anion transport	TIGR00886: 2A0108 nitrite extrusion protein (nitrite facilitator)
VC83_00102	ND	ND	GKZ77169.1	OWZ56854.1	1.11	4.72	Cytoplasm	electron transport	cl09933: ACAD Acyl-CoA dehydrogenase
VC83_05465	ERG11	ERG11	GKZ67210.1	OXG23736.1	1.64	4.39	Membrane	electron transport	cl12078: p450 Cytochrome P450
VC83_04555	ND	KHC76775.1	GLA40930.1	ND	-1.02	1.69	Membrane	electron transport	ND
VC83_08787	FRE5	CFL11	GKZ79616.1	OWT38774.1	-1.33	2.74	Plasma membrane	electron transport	cd06186: NADPH oxidase (NOX)
VC83_08518	JLP1	KHC83894.1	KAI3021159.1	UOH83015.1	1.84	5.65	Cytoplasm	electron transport	pfam02668: TauD Taurine catabolism dioxygenase
VC83_08524	DIT2	ERG5	KAI3009392.1	OWZ69196.1	1.61	5.03	ND	electron transport	cl12078: Cytochrome P450
VC83_08570	ND	KHC86328.1	GKZ67210.1	UOH82104.1	1.01	2.21	Membrane	electron transport	cl12078: p450 Cytochrome P450
VC83_08659	AIM17	KHC85949.1	EHA24645.1	OWZ80585.1	1.63	3.42	Mitochondrion	electron transport	cl26676:Taurine dioxygenase
VC83_08941	ERG11	ALK2	GKZ77838.1	OXB38448.1	1.00	8.13	ND	electron transport	cl12078: p450 Cytochrome P450
VC83_02428	FRE7	FRP1	EHA27596.1	OXG26069.1	1.26	7.00	Plasma membrane	electron transport	cd06186: NOX_Duox_like_FAD_NADP NADPH oxidase
VC83_02488	CBR1	CBR1	GAQ41792.1	OXC64979.1	1.98	5.11	ND	electron transport	cl26810:nitrate reductase [NADPH]
VC83_02494	YHB1	YHB1	GLA16543.1	OWT39490.1	1.95	4.89	Intracellular anatomical structure	electron transport	cl26811:NAD(P)H-flavin reductase
VC83_03470	ND	ND	GKZ82666.1	OWT41612.1	-1.13	6.52	Membrane	electron transport	cl27552: FAD_binding_3 Superfamily FAD binding domain
VC83_03003	ND	ND	EHA20505.1	OXC80805.1	1.25	6.21	NA	electron transport	cd04730:2-Nitropropane dioxygenase (NPD)
VC83_03096	FRE7	FRP1	KAL3253552.1	OWZ43078.1	-1.70	8.52	Plasma membrane	electron transport	cd06186:NADPH oxidase (NOX)
VC83_04399	ND	ND	EHA22966.1	ND	-1.22	5.77	Integral to membrane	electron transport	cd08760: Cyt_b561_FRRS1_like; Eukaryotic cytochrome b(561)
VC83_00191	ND	ND	TPR06291.1	ND	-4.70	7.65	Plasma membrane	copper ion transport	pfam04145:Ctr copper transporter family
VC83_08495	ND	SOD5	GKZ53660.1	ND	1.52	4.52	TransMembrane:1	superoxide metabolic process	pfam00080:Copper/zinc superoxide dismutase
VC83_00837	CTS2	Q5AKZ3.1	GLA07435.1	OWZ77110.1	1.12	4.40	NA	carbohydrate metabolic process	cl16916: ChtBD1 Hevein or type 1 chitin binding domain
VC83_08561	SGA1	SGA1	AIY23067.1	OXG32870.1	1.96	7.55	Vacuole	carbohydrate metabolic process	cd05811: CBM20_glucoamylase Glucoamylase

*ND-Not determined
